# Tiny Joints, Big Clues: Neonatal Septic Polyarthritis as a Rare Presentation of Late-Onset Streptococcus agalactiae Sepsis

**DOI:** 10.7759/cureus.88300

**Published:** 2025-07-19

**Authors:** Vijayalaxmi Tiwari, Karteeka Chichhal, Yasha Mukim, Raunak Bir, Pooja Pandey, Rajiv Gupta

**Affiliations:** 1 Microbiology, Employees' State Insurance Corporation (ESIC) Medical College and Hospital, Faridabad, IND

**Keywords:** b-lymphocyte depletion, gram-positive cocci, neonatal late-onset sepsis, pediatric septic arthritis, streptococcus agalactiae

## Abstract

Group B Streptococcus (GBS), or Streptococcus agalactiae, is a significant cause of neonatal morbidity and mortality, with late-onset disease (LOGBS) typically manifesting as bacteremia or meningitis. Osteoarticular infections, particularly septic polyarthritis, are exceedingly rare but potentially devastating complications of LOGBS. We report a rare case of LOGBS presenting as septic polyarthritis in a 28-day-old term male neonate. The infant presented with a 15-day history of progressive swelling in the left knee and right shoulder, accompanied by irritability and feeding difficulties. Ultrasonography revealed joint effusions, and aspiration yielded Gram-positive cocci in chains. Culture confirmed S. agalactiae, susceptible to ceftriaxone and vancomycin but resistant to multiple other antibiotics. Targeted antibiotic therapy was initiated, and surgical drainage of both joints was performed. The infant responded well, with complete clinical recovery. Immunophenotyping revealed reduced B-lymphocyte levels, suggesting a potential underlying immune vulnerability. This case underscores the importance of recognizing atypical manifestations of GBS in neonates. Prompt microbiological diagnosis using Gram stain and culture, combined with susceptibility-guided antimicrobial therapy and early surgical intervention, was instrumental in achieving a favorable outcome. The lymphocyte subset abnormality raises the possibility of an underlying immune dysfunction, warranting further follow-up. In regions without routine GBS screening, clinicians must maintain a high index of suspicion for uncommon presentations like septic polyarthritis. Early recognition, multidisciplinary management, and individualized therapy are key to optimizing outcomes in such rare neonatal infections.

## Introduction

Group B Streptococcus (GBS), or Streptococcus agalactiae, is a Gram-positive bacterium belonging to the Lancefield group B streptococci. Though commonly a harmless commensal in the gastrointestinal and genitourinary tracts of healthy adults, it can cause serious infections in neonates, pregnant women, the elderly, and immunocompromised individuals [1]. Vaginal or rectovaginal colonization occurs in 10-30% of pregnant women and is linked to complications such as urinary tract infections, chorioamnionitis, preterm labor, and stillbirth [2]. Since the 1970s, GBS has emerged as a major global cause of neonatal sepsis, pneumonia, and meningitis, contributing to 90,000 infant deaths annually, especially in low-income regions [2].

Neonatal sepsis is commonly classified into two types based on when symptoms begin: early-onset sepsis (EOS), which occurs within the first few days of life (usually within 72 hours), and late-onset sepsis (LOS), which develops after that period, typically up to 28 days of age [3]. The exact cutoff between the two can vary, with some experts using 72 hours and others using seven days as the dividing point. Neonatal GBS disease is clinically classified based on the timing of onset: early-onset disease (EOGBS), occurring within the first six days of life, and late-onset disease (LOGBS), presenting between seven and 90 days after birth. Infections occurring beyond 90 days, up to six months of age, are termed ultra-late-onset GBS disease (ULOGBS). Though ULOGBS and LOGBS are microbiologically and clinically similar, ULOGBS is more frequently associated with prematurity [4,5].

Bacterial septic arthritis is one of the emergency rheumatic conditions because of serious morbidities, permanent disabilities, and death, with mortality rates of 10-15% [6]. Delayed or inadequate treatment can lead to irreversible joint destruction and morbidities, which occur in about 25-50% of affected patients [7]. The main causative pathogens for septic arthritis are Staphylococcus aureus and Streptococcus species. Late-onset neonatal sepsis by GBS manifests as bacteremia without a focus (65%), meningitis (25%), and cellulitis and osteoarthritis (2-3%) [8]. Global incidence of neonatal septic arthritis is approximately 0.3 per 1000 live births, whereas in India, it has been reported as 0.6 per 1000 live births [9]. The incidence of GBS infection is only 0.17 per 1000 live births, highlighting the rarity of GBS as an important pathogen causing late-onset sepsis in this context. The estimated incidence of EOGBS and LOGBS were 0.58/1000 live births (95% CI 0.46- 0.73) and 0.33/1000 live births (95% CI 0.24- 0.45), respectively. The overall mortality was 7% [5]. Routine GBS screening is not currently practiced in India due to logistical challenges, financial constraints, and the high number of home births.

## Case presentation

We present a case involving a male neonate born at term (40 weeks of gestation) to a primigravida mother following an uneventful pregnancy and normal vaginal delivery. The infant had a birth weight of 3110 grams and demonstrated robust neonatal well-being, with Apgar scores of 9 and 10 at one and five minutes, respectively. There was no history of prolonged rupture of membranes, and the perinatal period was uneventful.

**Figure 1:**
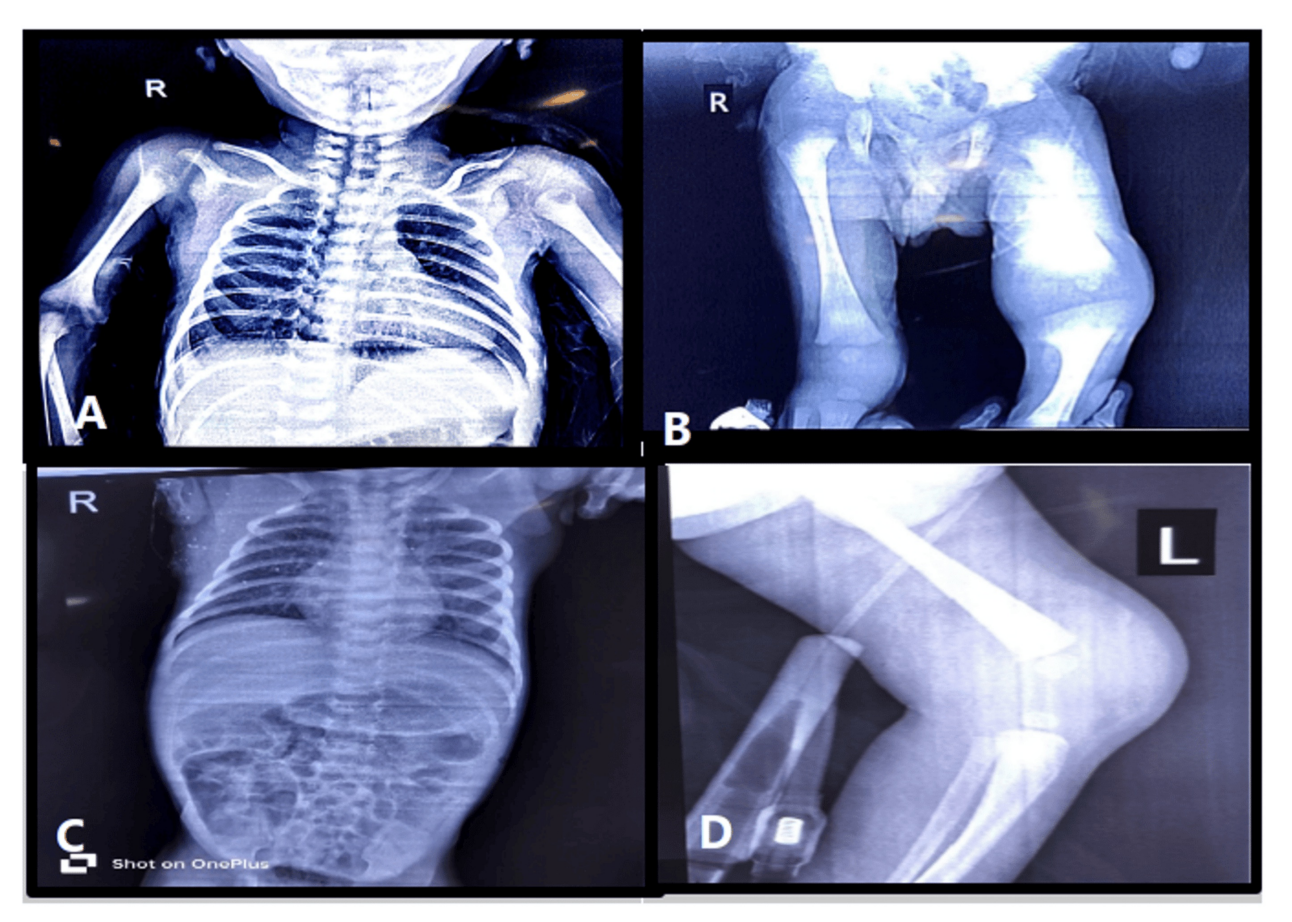
A: AP view showing fluid collection in the right shoulder, B: AP view showing fluid collection over the left knee, C: AP (abdomen) view showing fluid collection in the right shoulder, and D: AP view showing negative suctioning drain in the left knee

On the 28th day of life, the infant was brought to the emergency department with a 15-day history of progressive swelling in the left knee followed by swelling in the right infra-axillary region, accompanied by persistent crying and visible discomfort during limb movement. On clinical examination, the neonate appeared lethargic, irritable and refused feeds. Local examination revealed inflamed, tender swellings at both affected sites. An X-ray of the chest and abdomen was also obtained (Figure 1).

Ultrasonography (USG) demonstrated a fluid collection of 6-7 mL in the deeper planes of the right shoulder, while the left knee exhibited a joint effusion of 15mL with internal echoes. USG-guided aspiration was performed at both sites, and the aspirated samples were sent for cell count, fluid biochemistry along with aerobic culture and antimicrobial susceptibility testing before initiating empirical antibiotic therapy.

The fluid was turbid with >90,000 cells/mm^3^ predominantly neutrophils, with increased protein and decreased sugar suggesting an underlying bacterial infection.

**Figure 2:**
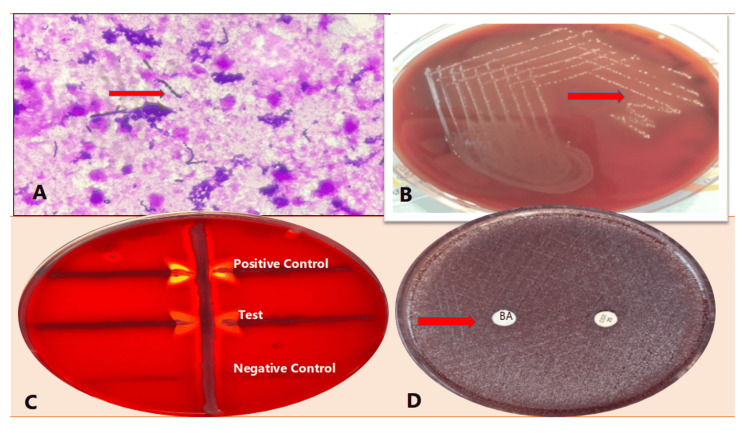
A: Plenty of pus cells and Gram-positive cocci in pairs and short chains observed from the direct sample, B: Beta hemolytic colonies on a blood agar plate, C: Enhanced zone of hemolysis seen on the CAMP test as arrowheads, and D: Blood agar plate showing resistance to bacitracin and co-trimoxazole

Gram staining of the aspirate revealed numerous pus cells and Gram-positive cocci arranged in pairs and chains (Figure 2A). These findings were promptly communicated to the attending clinician, who initiated intravenous cefotaxime and a renal-adjusted dose of vancomycin. Primary culture of the aspirates was performed under aseptic conditions and inoculated plates were incubated aerobically and in microaerophilic conditions at 37°C. After 24 hours, beta-hemolytic colonies were observed on blood agar (Figure 2B), which were catalase-negative. For further confirmation, a CAMP test was done, which was read post 18-24 hours and showed a positive result (Figure 2C). Bacitracin and Co-trimoxazole disc susceptibility was also performed for species identification which showed resistance to both (Figure 2D).

Further identification and antimicrobial susceptibility testing were conducted using the automated VITEK-2 ID/AST system (BioMérieux). Within 16-18 hours, the causative organism was confirmed as Streptococcus agalactiae (Group B Streptococcus) exhibiting susceptibility to benzylpenicillin, ceftriaxone, vancomycin, and linezolid, but resistance to gentamicin, tetracycline, chloramphenicol, erythromycin and clindamycin. Based on the susceptibility profile, vancomycin was discontinued, and cefotaxime was further continued.

As part of the comprehensive evaluation, flow cytometric immunophenotyping of lymphocyte subsets was performed which showed a reduction in B-lymphocytes and increased T-lymphocytes (Table 1).

**Table 1:** Flow cytometric immunophenotyping of lymphocyte subsets Impression: B-lymphocytes are reduced in number with an increase in T-lymphocytes

Lymphocyte subset	Patient	Normal range
Absolute lymphocyte count	1,633/µL	1900-3700/cmm
Percent of CD3+(T lymphocyte)	80.83%	60-76%
Percent of CD4+ (T Helper lymphocytes)	73.37%	30-60%
Absolute CD4+ count	968/ µL	500-1500 cells/µL
Percent of CD8+ (T Cytotoxic lymphocytes)	21.86%	12 – 40%
Absolute CD8+ count	288/ µL	300 – 1,000 cells/µL
CD4:CD8 Ratio	3.36:1	1.5-2.5:1
Percent of CD19+ (B lymphocytes)	6.58%	13-27%
Percent of CD56+ (NK lymphocytes)	9.8%	04-13%

The flow cytometric immunophenotyping revealed reduced B-lymphocytes and increased T-lymphocytes. The surgical procedures, left knee arthrotomy and right shoulder incision and drainage, were performed under GA using strict aseptic conditions. The neonate demonstrated significant clinical improvement and was discharged after 23 days of inpatient care. The neonate in our case demonstrated full clinical recovery and is presently on weekly follow-up.

## Discussion

This case report describes a rare presentation of LOS caused by GBS manifesting as septic polyarthritis involving the left knee and right shoulder region in a term neonate. This is the first documented case of neonatal septic polyarthritis due to GBS presenting as late-onset sepsis at our institution. While GBS is a well-recognized cause of neonatal sepsis, focal infections like osteoarticular involvement (OAI) represent a smaller percentage of LOS cases, typically reported as 2-3% for cellulitis and osteoarthritis combined. Polyarticular involvement, as seen in this patient, is even less common. Septic arthritis is generally monoarticular, though polyarticular forms can occur, especially in neonates due to hematogenous seeding [10,11]. A study by Schuler et al. (2021) reported septic arthritis as a rare presentation of GBS LOD, contributing to only 4% in an Italian cohort and 0.73% in a Japanese cohort of all LOD cases, emphasizing its rarity [12]. Another review notes that while Staphylococcus aureus is the most frequent pathogen in OAI in infants under three months, GBS is becoming more prevalent in some regions, even after labor prophylaxis [13].

The diagnosis of septic arthritis in neonates can be challenging due to non-specific symptoms such as irritability, refusal to feed, and pseudoparalysis of the affected limb(s) [12,14]. In this case, the 15-day history of progressive swelling and discomfort guided the clinical suspicion. Ultrasonography proved invaluable for confirming joint effusion and soft tissue collection, and for guiding aspiration, which is crucial for both diagnosis and management. Early diagnosis and intervention are critical to prevent devastating sequelae [10,14].

A critical aspect highlighted by this case is the paramount importance of early and accurate microbiological diagnosis. The initial Gram stain findings of Gram-positive cocci in chains from the aspirated fluid were instrumental in guiding appropriate empirical antibiotic therapy. Subsequent culture confirmation of S. agalactiae and its susceptibility profile allowed for de-escalation of antibiotics, discontinuing vancomycin and continuing with ceftriaxone, aligning with antimicrobial stewardship principles. The observed resistance to erythromycin and clindamycin underscores the necessity of susceptibility testing.

The timely surgical intervention, involving arthrotomy of the left knee and right shoulder incision and drainage, was crucial in managing the septic foci and preventing long-term complications such as joint destruction, limb deformity, and growth impairment. Surgical drainage is commonly recommended for septic arthritis to obtain biological samples and decrease intra-articular pressure [10,11]. The multidisciplinary approach was instrumental in the favorable outcome.

An interesting finding in this case was the altered lymphocyte subset profile, with a reduction in B-lymphocytes (6.58%) and an increase in T-lymphocytes (CD3+ 80.83%). While neonatal immune responses can be variable, a significant reduction in B-lymphocytes could imply a transient or underlying humoral immune deficiency, which might have predisposed the infant to severe GBS infection or LOGBS. Although specific case reports directly linking B-lymphocytopenia to neonatal GBS polyarthritis are scarce in the initial search, GBS infections, particularly late-onset and recurrent ones, have been associated with immune defects. Further immunological evaluation during follow-up is warranted.

This case is reported in India, where routine maternal GBS screening and intrapartum antibiotic prophylaxis are not standard practice. Consequently, clinicians must maintain a high index of suspicion for GBS infections, including less common manifestations like septic polyarthritis, in neonates presenting with signs of sepsis or localized inflammation. The incidence of LOGBS may be underrecognized in such settings. A review on neonatal GBS infections in India highlighted that despite significant maternal colonization rates, reports of invasive neonatal GBS disease were infrequent, possibly due to a large number of home births and underreporting, suggesting the true incidence is largely unknown [15].

## Conclusions

This case highlights a rare occurrence of septic polyarthritis due to GBS as a manifestation of late-onset neonatal sepsis in a term infant. It underscores the critical importance of early clinical suspicion, rapid microbiological evaluation including Gram stain and culture, and targeted antimicrobial therapy based on susceptibility results. Furthermore, timely and effective surgical drainage of septic foci is paramount in preventing devastating long-term sequelae associated with neonatal septic arthritis.

The immunological finding of reduced B-lymphocytes warrants further investigation during follow-up. In a country like India, where routine GBS screening is not universally implemented, heightened awareness and a high index of suspicion for GBS are essential for the early diagnosis and management of such severe neonatal infections. This case reinforces that a prompt, multidisciplinary approach can lead to favorable outcomes even in rare and severe presentations of neonatal GBS disease.
